# Beyond the Heart: Hepatic Extracellular Volume Assessment in Patients With Cardiovascular Disease

**DOI:** 10.1002/jmri.70452

**Published:** 2026-07-14

**Authors:** Kenichiro Suwa, Yuichiro Maekawa

**Affiliations:** ^1^ Division of Cardiology, Internal Medicine III Hamamatsu University School of Medicine Hamamatsu Japan

**Keywords:** cardio‐hepatic axis, cardiovascular disease, hepatic ECV, magnetic resonance imaging, myocardial infarction

Cardiac magnetic resonance imaging (MRI) has added quantitative tissue characterization, particularly T1 mapping and extracellular volume (ECV) fraction quantification, to cardiovascular imaging. These techniques, initially developed to assess myocardial fibrosis and interstitial expansion, are central to evaluation of cardiomyopathies, myocardial inflammation, and infiltrative diseases. Routine cardiac MRI frequently extends beyond the myocardium to include extracardiac structures, including the liver. Traditionally considered incidental, the liver may provide insights into systemic remodeling that occurs in cardiovascular disease. Growing evidence suggests an association and bidirectional pathophysiological influence between heart and liver.

Hepatic ECV is a quantitative surrogate for interstitial expansion and fibrosis. MRI‐based hepatic ECV correlates with histologic fibrosis stage in chronic liver disease, including metabolic dysfunction‐associated steatotic liver disease, and associates with established clinical markers including Child–Pugh class and portal hypertension [1, 2]. Comparisons with magnetic resonance elastography further support ECV's relationships with liver stiffness and extracellular matrix remodeling. Translating hepatic ECV from hepatology to cardiology introduces interpretive complexity. In patients undergoing MRI for cardiac indications, elevated hepatic ECV may reflect chronic fibrosis but may also capture acute or chronic hemodynamic perturbations. Increased central venous pressure, systemic inflammation, neurohormonal activation, and extracellular matrix remodeling following acute myocardial infarction (MI) may contribute to hepatic interstitial expansion.

Recent studies support the relevance of hepatic imaging biomarkers in cardiovascular disease patients. In right‐sided heart failure with Fontan circulation, chronic systemic venous congestion is associated with elevated hepatic T1 and ECV levels, reflecting progressive liver remodeling secondary to longstanding hemodynamic stress [3]. Similarly, in left‐sided heart failure with dilated cardiomyopathy, hepatic T1 mapping parameters have been associated with adverse outcomes, suggesting that the liver may serve as a surrogate marker for systemic congestion and chronic low‐output states [4]. In systemic infiltrative diseases including amyloidosis, multiorgan ECV expansion further highlights the interconnected nature of extracellular remodeling [5]. Together, these observations suggest that hepatic ECV in cardiovascular disease may function less as an organ‐specific fibrosis marker and more as an integrative indicator of systemic stress. Although this broader interpretation reduces mechanistic specificity, it enhances the potential value of hepatic ECV as a biomarker of global cardiovascular risk.

Recent observations in patients with MI have extended this concept. Hepatic T1 and ECV measurements obtained during routine cardiac MRI appear to provide prognostic information beyond conventional cardiac imaging parameters and clinical risk factors [6]. These findings suggest that hepatic tissue characteristics after myocardial injury may capture systemic vulnerabilities that are not fully reflected by myocardial assessment alone. Several mechanisms can explain this association. Acute MI induces inflammatory activation and neurohormonal responses that influence extracellular matrix remodeling beyond the myocardium [7]. Transient or persistent elevations in venous pressure due to ventricular dysfunction may contribute to hepatic congestion and interstitial expansion [8]. Hepatic abnormalities may also reflect preexisting systemic metabolic and fibrotic burdens rather than cardiac injury alone. Recent evidence indicates that liver fibrosis is associated with increased risk of coronary artery disease and adverse cardiovascular outcomes, supporting the premise that hepatic remodeling may serve as a marker of systemic cardiovascular vulnerability [9]. Consistent with this premise, hepatic T1 obtained from routine cardiovascular magnetic resonance has been shown to predict cardiovascular risk in a broad clinical population, suggesting that liver tissue characteristics may provide incremental prognostic information beyond conventional cardiac assessments [10].

The broader implication supports a shift from organ‐focused imaging to multiorgan phenotyping. The liver, which is routinely visualized during cardiac MRI but rarely analyzed, may provide complementary information regarding congestion, inflammation, and systemic remodeling. As hepatic ECV can potentially be derived from existing cardiac MRI protocols without additional scanning time, it is an attractive candidate for integration into future cardiovascular risk assessment strategies.

Future research should focus on standardizing hepatic ECV acquisition and analysis, validating its associations with clinical outcomes in diverse populations, and clarifying its biological relationships with established markers, including elastography, circulating biomarkers, and histopathological findings. Longitudinal studies are warranted to determine whether changes in hepatic ECV are associated with disease progression, recovery, or response to therapy.

As cardiovascular imaging continues to evolve, informative signals may not be confined to the heart. Liver information captured during routine cardiac MRI may provide insights into systemic vulnerability and long‐term risks.
